# Investigation into the electrochemical behaviour of silver in alkaline solution and the influence of Au-decoration using *operando* Raman spectroscopy

**DOI:** 10.1039/c9ra10282e

**Published:** 2020-02-26

**Authors:** Haowei Luo, Xu Ji, Shuang Cheng

**Affiliations:** Guang Zhou Key Laboratory for Surface Chemistry of Energy Materials, School of Environment and Energy, South China University of Technology Guangzhou 510006 P. R. China escheng@scut.edu.cn; College of Automation, Zhongkai University of Agriculture and Engineering Guangzhou 510225 China

## Abstract

To explore the basic chemistry in the electrochemical environment, the electrochemical behavior of Ag and the influence of Au decoration is investigated with cyclic voltammetry (CV), galvanostatic charge–discharge (GCD) and *operando* Raman measurements in a 1 M KOH solution. During the anodic CV sweep, Ag is oxidized to Ag_2_O in the first step through a one-electron process, and then, AgO in the second step through another one-electron process. Meanwhile, some AgO is formed at a relatively low potential under the irradiation of visible lights (photoelectrochemical oxidation). In the GCD mode, it is found that apart from the two one-electron processes, part of the Ag is oxidized to AgO directly through a two-electron process in the second oxidation step, implying slightly different activities of these reactions in the CV and GCD mode. During cathodic CV sweep and galvanostatic discharge, opposite reactions take place respectively. The coulombic efficiency is calculated to be only ∼82% from the CV cycle at 5 mV s^−1^ due to the formation of silver hydroxyl species (oxidation state) in a low potential range. For the Au decorated Ag, Raman signals from these species disappeared and the coulombic efficiency is enhanced to 95%, indicating an obvious improvement in reversibility.

## Abstract 2

Apart from the traditional charge storage mechanism demonstrated in CV, a two-electron process of Ag to AgO is revealed in GCD.

## Graphical abstract

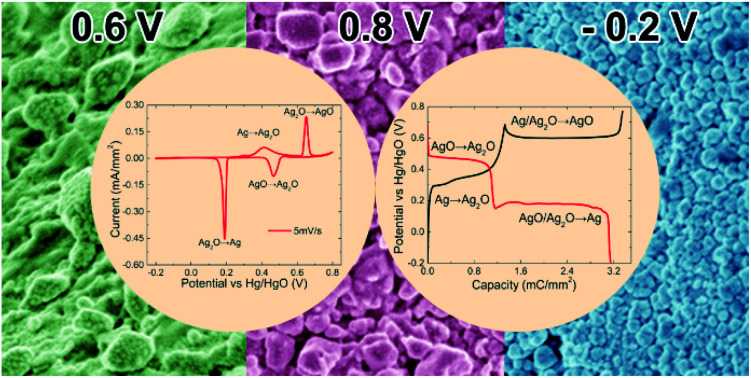

## Introduction

With the increasing demand for energy in the human society, the development of renewable energy has been promoted in the past few decades, with a variety of energy storage devices including supercapacitors and rechargeable batteries.^1–4^ Supercapacitors have been applied to various fields such as sustainable energy collection (*e.g.* wind and tidal energy) and electric automobiles because of their high power density.^5,6^ However, their widespread use has been limited by low energy density. Batteries, especially lithium-ion batteries, are famous for their high energy density, while their rate performance and safety are always unsatisfied. The construction of aqueous-based batteries is one of the effective approaches to balance the power and energy density as well as safety. The exploration of suitable electrodes that can be used in aqueous systems is critical, including anodes and cathodes.

Ag, famous for its use in SERS^7^ due to the existence of surface plasmon resonance (SPR),^8–10^ has been well considered as cathodes for alkaline solutions in recent years, especially in zinc–silver (Zn–Ag) batteries,^11,12^ due to its high capacity and high operational potential. Ag–Zn battery is one of the promising aqueous batteries that possesses high energy density owing to the high capacities for both electrodes and a considerable output voltage. However, when used as a cathode in a battery, there were problems, including the low coulombic efficiency caused by incomplete reversible reactions and the low power density owing to the use of un-conductive polymer binders and the high resistance of the middle product Ag_2_O, which have greatly hindered its further development. To address these issues, binder-free electrodes have been explored.^13^ Besides, the composition of nanostructured noble metals (such as gold and platinum) has also been considered as a promising approach because of their high electronic conductivity and high inertia.^14^ However, to figure out a possible solution to the problems aroused by the reaction of Ag, its electrochemical behavior needs to be thoroughly investigated.

Commonly, the faradic reactions of silver involve two redox reaction steps at equilibrium potential corresponding to a one-electron-transfer process for each step as follows:^12^1
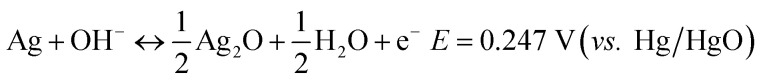
2
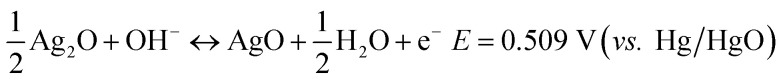


Eqn (1) stands for the first oxidation step from Ag to Ag_2_O, at operation potential ∼ 0.35 V *vs.* Hg/HgO during the anodic sweep, which is a diffusion-limited process. The later equation corresponds to the second oxidation step at operation potential ∼ 0.7 V *vs.* Hg/HgO where Ag(i) is further oxidized at a higher valence of Ag(ii), which is a nucleation and growth process. The theoretical capacity of Ag is up to 893 C g^−1^ for a one-electron-transfer process, which should be doubled for the two reaction steps. However, the practical capacity is often far below this value when Ag is used as the cathode in a battery.^15,16^

Although the electrochemical behavior of silver in alkaline solution has been discussed in previous reports,^17,18^ these studies are not systematic and only the two reaction steps were discussed with cyclic voltammetry (CV) scans. Most importantly, no solutions have ever been proposed towards the irreversible reactions as well as the low coulombic efficiency. Herein, the behavior of Ag in 1 M KOH is fully investigated using CV, galvanostatic charge–discharge (GCD) and *operando* Raman analysis during the CV cycling. It is found that, except for the traditional reaction mechanism discussed above, there is a two-electron process during the second reaction step in the GCD mode. Furthermore, a possible approach is proposed to enhance the reversibility of the faradic reactions.

## Results and discussion

### Electrochemical performance analysis

**Fig. 1:**
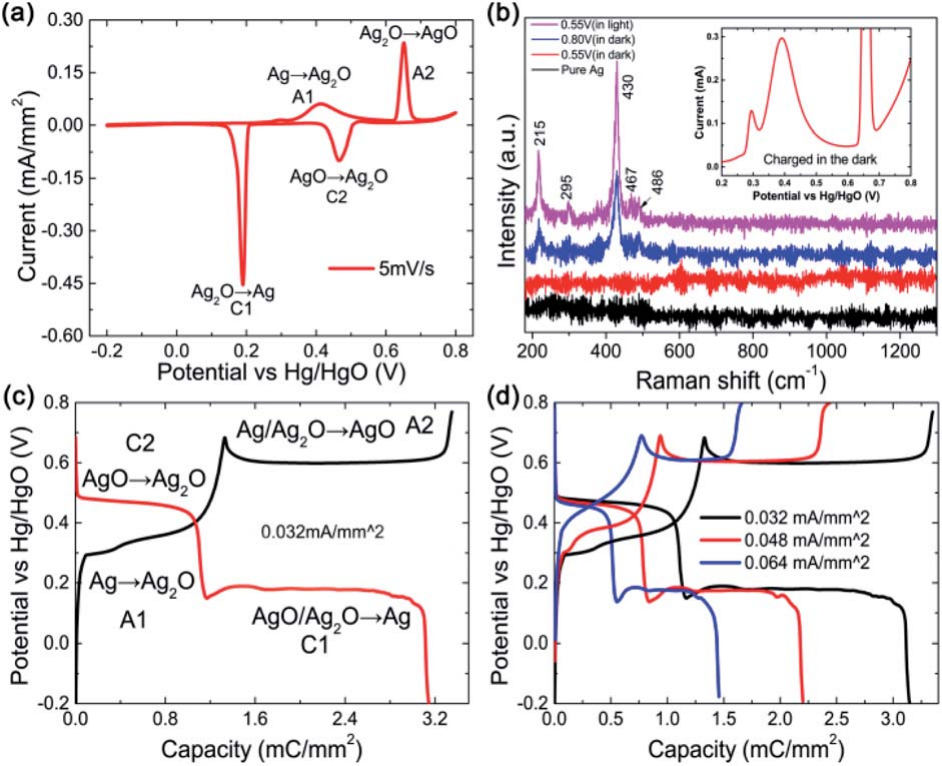
(a) Typical CV curve at 5 mV s^−1^. (b) *Ex situ* Raman spectra of the Ag wire collected at different potentials during the first anodic sweep running in dark or in natural light: black curve, pristine Ag; red curve, stopped at 0.55 V and blue curve, stopped at 0.8 V running in dark; magenta curve, stopped at 0.55 V running in natural light; inset, enlarged part CV curve charging from 0.2 V to 0.8 V. GCD curves (c) at a current density of 0.032 mA mm^−2^ and (d) at various current densities.

Electrochemical tests were conducted in a three-electrode system using a 1 M KOH aqueous solution as the electrolyte and Hg/HgO as the reference electrode, as shown in Fig. 1. Fig. 1a exhibits the general CV curve at 5 mV s^−1^. Two obvious oxidation peaks named as A1 with a front peak in the range of 0.25–0.5 V and A2 centred at ∼0.65 V can be observed at the anodic sweep curve. The A1 peak concerns the oxidation of Ag to Ag_2_O and the A2 peak should correspond to the further oxidation of Ag_2_O to AgO. In fact, the A1 peak together with its front peak is composed of 3 peaks.^18^ The front peak is assigned to the oxidation of Ag to Ag_2_O in the superficial layer and the latter two (looks like one peak in this work) are in the layers below.^19,20^

To confirm the reaction step mentioned above, *ex situ* Raman spectroscopy measurements were performed on the samples in different situations, as shown in Fig. 1b. No Raman bands arising from Ag_2_O can be observed in any of the spectra due to the high light sensitivity of Ag_2_O.^21^ In addition, there are no Raman bands for the electrode after the anodic sweep to 0.55 V (ending of the A1 peak) in dark (see the red curve in Fig. 1b), indicating that the A1 peak corresponds to the formation of Ag_2_O. Five bands at 215, 295, 430, 467 and 486 cm^−1^ that can be assigned to AgO emerge after the anodic sweep to 0.8 V (ending of the A2 peak, see the blue curve), indicating that the A2 peak corresponds to the formation of AgO. However, for the electrode tested in natural light, obvious Raman bands arising from AgO after the anodic sweep to 0.55 V can be observed (see the magenta curve), which is attributed to the photoelectrochemical oxidation of Ag to AgO in a low potential range.^22^ From the partially enlarged CV curve collected in the dark (inset of Fig. 1b), it can be seen that except for the two oxidation peaks of A1 and A2, the front peak of A1 is also observable, which means that there is no difference for the CV curves collected in darkness and in natural light, implying that only a little amount of photoelectrochemical reaction induced AgO is formed.

Reversible reactions occur during the cathodic sweep and two reduction peaks (named C2 and C1) are observed, which should correspond to the reduction of AgO to Ag_2_O and Ag_2_O to Ag, respectively. The coulombic charge passed by for each reaction can be calculated from the integration of the corresponding CV peak area, as presented in Table 1. It can be seen that the charges passed though peaks A1 and C1 are much larger than those of peaks A2 and C2, indicating that only a part of Ag_2_O is oxidized to AgO during the second oxidation step. The total charge transfer during reduction is less than that during oxidation and the coulombic efficiency is about 82%, which means that the reactions are not fully reversible.

**Table 1:** Digital values calculated from the GCD and CV results in Fig. 1 and 3

Anodic/cathodic peaks		A1	A2	C2	C1
CV capacity (mC mm^−2^)	Pristine Ag	2.06	1.28	0.8	1.95
	Treated Ag	6.45	5.17	3.79	7.19
GCD capacity (mC mm^−2^)	Pristine Ag	0.35	2.40	0.32	2.17
	Treated Ag	2.16	5.17	1.75	5.46

To further investigate the electrochemical behavior of Ag, GCD measurements were performed at 0.032 mA mm^−2^, as shown in Fig. 1c. Two obvious plateaus at ∼0.3 V and 0.6 V corresponding to the two-step oxidation process can be seen in the charge curve (dark line) and the reverse process can be observed in the discharge curve, which is consistent with the CV result. Similarly, two unusual phenomena are worth mentioning. One is the super long plateau for the second charge step, much longer than that for the first step, indicating much more charge-transfer during the second step, which is quite different from the CV result. The other one is a sudden potential drop in the second plateau during charge (A2). As is well known, the charge passed during the second oxidation step (Ag_2_O to AgO) should be less than that during the first step. One of the reasonable explanations is the direct oxidation of Ag to AgO through a two-electron process in the second step, which is not obvious in a potential continuous rising process of CV, implying a slightly different reaction process in the GCD mode and CV mode. The sudden potential drop at the beginning of the A2 and the sudden increase at the beginning of the C1 plateaus should be related to the high resistance of Ag_2_O (Table 2). When the Ag_2_O formed at the first oxidation step covered the surface of Ag, the charge potential should be high, while it would be reduced along with the formation of more conductive AgO. Moreover, the GCD curves at various currents display similar profiles (Fig. 1d), indicating that the reaction process is decided mainly by the current mode, constant or not, but not by the magnitude.

**Table 2:** Resistivity and density of Ag, Ag_2_O and AgO

Substance	Ag	Ag_2_O	AgO
Resistivity (Ω cm)	1.59 × 10^−6^	10^8^	10–15
Density (g cm^−3^)	10.9	7.15	7.44

**Fig. 2:**
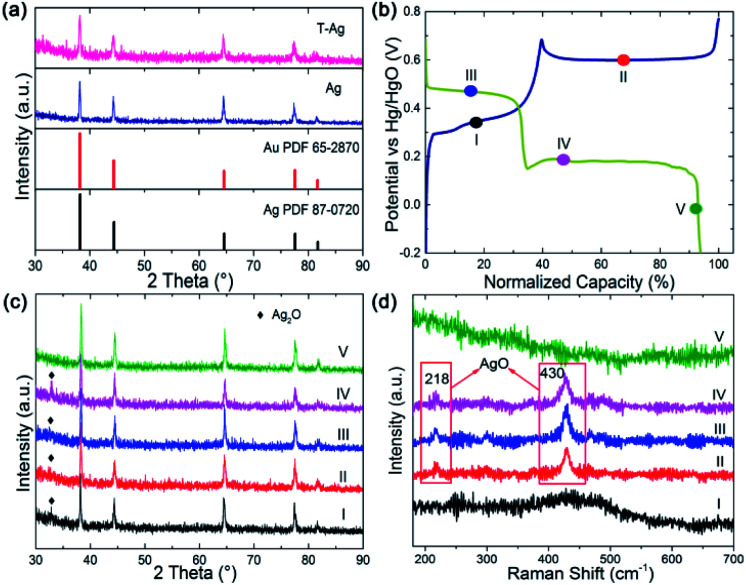
(a) XRD of Ag and Au modified Ag wires. (b) GCD curve of Ag for *ex situ* XRD and Raman measurement. (c) XRD pattern and (d) Raman spectrum collected at point I to V in (b).

To confirm the direct reaction of Ag to AgO, *ex situ* X-ray diffraction (XRD) patterns and Raman spectra were collected at different points (I to V) during the GCD, as shown in Fig. 2b–d, because Ag_2_O is detectable with XRD while AgO is sensitive to Raman spectroscopy. Ag_2_O, but not AgO, is detected at point I, indicating that only Ag_2_O is formed during the first oxidation step. The clear Raman signal that can be assigned to AgO emerged at points II, III and IV, implying that AgO is formed in the second oxidation step and it is not fully reduced after the first reduction step. AgO was left at the beginning of the second reduction step, while it disappeared at the end of this step and only Ag could be detected (reflected by the Raman and XRD result for point V). From these results, it can be clearly seen that AgO is directly reduced to Ag through a two-electron process during the second reduction step. The direct reaction of AgO to Ag or Ag to AgO can avoid the formation of high resistance Ag_2_O, which offers a promising strategy to resolve the rate issue of Ag-based chemical cells.

**Fig. 3:**
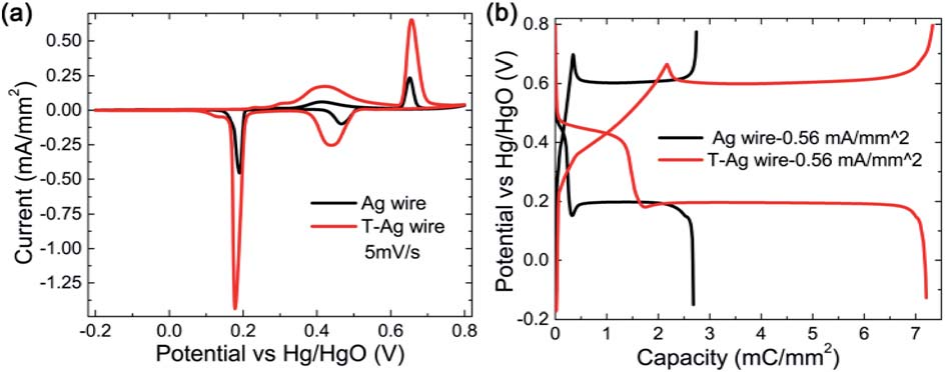
(a) CV and (b) GCD curves of pristine Ag and the T-Ag and at a same sweep rate and a same charge/discharge current density, respectively.

After being treated (modified with Au and part of the Ag on the surface is replaced by Au atoms, the sample is named as T-Ag), the CV and GCD profiles have no obvious changes compared with those of the pristine Ag sample (Fig. 3); the amount of Au is undetectable from XRD patterns (Fig. 2a). Meanwhile, it can be clearly seen that T-Ag exhibits much larger redox peaks than pristine Ag with the same sweep rate and geometric area (see Fig. 3a), which should result from the roughened surface and enhanced conductivity after the modification of Au. There are no reaction peaks related to Au, indicating that Au is stable during the electrochemical test.

From the digital values on the charge passed by, as shown in Table 1, it can be calculated that the coulombic efficiency is enhanced to 95% from 82% for the CV scan mode, indicating an improvement in the redox reactions' reversibility. In detail, the ratio of the Ag_2_O that is oxidized to AgO in the second reaction step as well as the ratio of the reverse reaction where AgO is reduced to Ag_2_O in the first discharge step is largely enhanced. For the GCD mode, the coulombic efficiency is also improved to 98% from 91% after modification.

### Morphology characterization

**Fig. 4:**
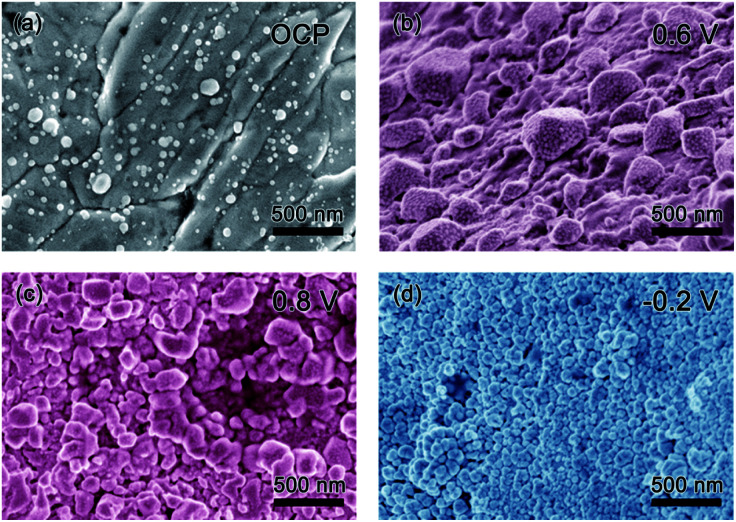
SEM images of the pristine Ag sample before CV cycling (a) and along CV cycling when the sweep potential stopped at different position: anodic sweep (b) 0.6 V and (c) 0.8 V, cathodic sweep (d) −0.2 V.

Scanning electron microscopy (SEM) was used to explore the morphology change of the pristine Ag sample along with the CV cycling under natural light (Fig. 4). Before testing with an open circuit potential (OCP), the surface of the sample is not smooth with some particles distributed on it, as presented in Fig. 4a. Along with the anodic sweep, after the first step corresponding to the oxidation of Ag to Ag_2_O (stopped at 0.6 V), Ag_2_O particles are formed with different sizes in the range of tens to hundreds of nanometers (Fig. 4b), which further confirms the prior statement that the oxidation of Ag to Ag_2_O is controlled by a diffusion process.^18^ Besides, there are few very small nanoparticles on the surface of the large Ag_2_O particles, which should be the photoelectrochemical product of AgO because it is reported that its formation is controlled by nucleation and growth processes and hence its size should be relatively uniform. Seen from the image collected after the second oxidation step (stopped at 0.8 V), AgO particles of relative uniform size are formed, as presented in Fig. 4c. When the potential is stopped at −0.2 V in the cathodic sweep during the reduction process, Ag nanoparticles with a uniform size of several tens of nm are formed (Fig. 4d). The results coincide with the prior report,^18^ which defends that the oxidation of Ag_2_O to AgO and the reduction of Ag_2_O to Ag are controlled by nucleation and growth processes. Meanwhile, it can be seen that CV cycling is a good way to prepare Ag nanoparticles for SERS research.^23^

**Fig. 5:**
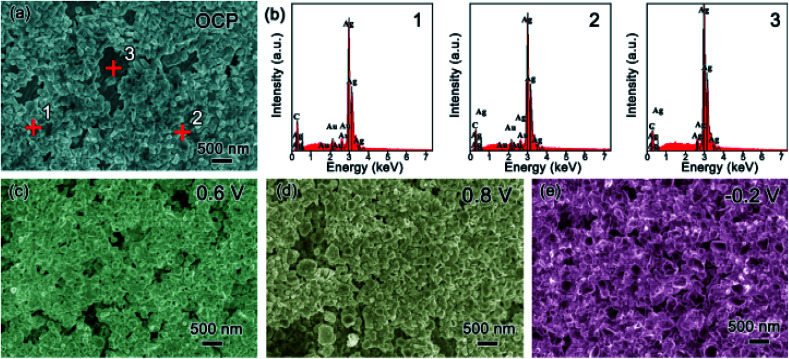
Characterization to the T-Ag sample: (a) SEM image at OCP and (b) EDS of different positions pointed in (a) (1 and 2 for decorated area and 3 for bare area); SEM images along CV and stopped at different potential (c) 0.6 V and (d) 0.8 V during the anodic sweep and (e) −0.2 V during the cathodic sweep.

To explore the effect of Au decoration, the surface change of the T-Ag along cycling was also recorded in Fig. 5. After decoration at OCP, most of the Ag surface is covered by particles, as shown in Fig. 5a. From the energy dispersion spectra (EDS, Fig. 5b) of three different positions pointed in Fig. 5a, the particles formed on the surface are mainly Au while the bare surface remains Ag. During the anodic CV sweep under natural light, larger particles are formed among the Au particles at 0.6 V (Fig. 5c), which should be the high resistant Ag_2_O. Because of the existence of Au particles, the continuous distribution of Ag_2_O is broken to some extent. As the potential is increased to 0.8 V, the inhomogeneity of the particles increases (Fig. 5d). During the cathodic sweep, when the potential is reduced to −0.2 V, Ag nanoparticles with a smooth surface and the smaller size are formed (Fig. 5e). Although it is difficult to distinguish Au particles from Ag or AgO_*x*_ ones, it can be speculated that Au particles remain among these species during the cycling and benefit from the fast electronic transport of the electrode.

### *In situ* Raman spectroscopy analysis

**Fig. 6:**
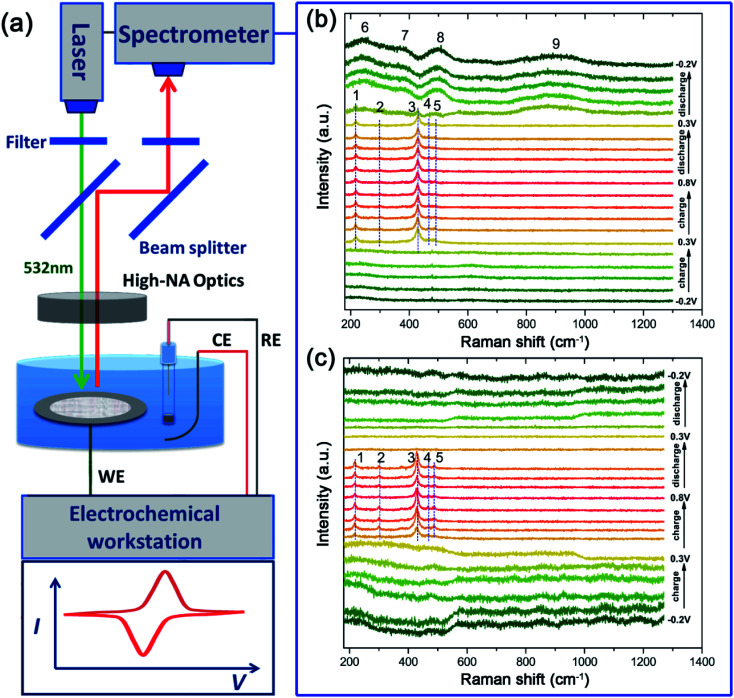
(a) Schematic of the equipment for *operando* Raman spectroscopy and the *operando* Raman spectra of the pristine silver electrode (b) and the T-Ag electrode (c) collected every 160 seconds during a CV cycle at a sweep rate of 0.625 mV s^−1^.

To confirm the oxidation steps discussed above, *operando* Raman spectroscopy measurement (Fig. 6) is employed to monitor the change of the surface species during the charge/discharge process. One spectrum is recorded every 160 seconds and a total of 21 spectra can be obtained in one CV cycle in a potential range of −0.2 V to 0.8 V at a sweep rate of 0.625 mV s^−1^. The corresponding Raman spectra obtained are presented in Fig. 6b. Almost no Raman bands can be observed between −0.2 V and 0.2 V during the anodic sweep, suggesting that Raman active oxides are not formed. Then, five bands (named as bands 1 to 5) that can be indexed to AgO^24^ are observed at 0.3 V, which are maintained in the following anodic sweep till the potential rises to 0.8 V. As discussed above, AgO should be formed at a higher potential of ∼0.7 V. The AgO signal observed at 0.3 V originates from the photoelectrochemical oxidation of Ag to AgO.^22^ The CV cycling is run under Raman laser irradiation without natural light during *operando* Raman measurements, which means that the photoelectrochemical oxidation can also be induced by Raman laser (532 nm used here).

During the anodic sweep, the intensity of the main band at ∼430 cm^−1^ is the strongest at ∼0.3 V while there is only a small amount of AgO, which should be caused by the SERS effect of the rough Ag surface^25,26^ at a low potential range before thick Ag_2_O is formed. However, during the cathodic sweep from 0.8 V to 0.2 V, the AgO signal is maintained during this whole range, implying that the reduction of AgO lasts over a wide potential range accompanied by a direct reduction of AgO to Ag at ∼0.2 V. When the potential declines further, below 0.2 V, four wide Raman bands (bands 6 to 9) emerge, which can be ascribed to the silver hydroxide (Ag(OH)_*x*_^*n*−^) that should be formed from Ag_2_O.^18^ These bands are still clearly visible even when the potential is decreased to −0.2 V, which should be the main reason for the lower practical capacity of silver than its theoretical value.

To explore the effect of Au decoration on the faradic reactions, *operando* Raman analysis was also employed for the treated sample of T-Ag, as shown in Fig. 6c. It can be clearly seen that the existence of AgO is much shorter than that of bare Ag, which means that the threshold potential of the photoelectrochemical oxidation of Ag to AgO increases slightly, and the full reduction of AgO is finished at higher potential (all of the AgO is reduced at the first reaction step that corresponds to the first reduction peak), indicating a much higher reversibility and reduced polarization.

Besides, the intensity change tendency is different from that of the bare Ag sample, which may be related to the increase in the photoelectrochemical oxidation potential (higher than the oxidation potential of Ag to Ag_2_O). Ag_2_O is formed before the formation of AgO, and hence, the SERS effect of Ag to AgO is hindered.

Another change that is worth mentioning is that there is no obvious Raman band in the cathodic sweep range of 0.4 V to −0.2 V after the disappearance of AgO, implying that there are no irreversible products (oxidized Ag) after the cathodic sweep. The decoration of only a little Au can largely enhance the efficiency and reversibility of the faradic reactions of Ag in an alkali based solution.

## Experimental

### Sample preparation

Silver wires (3N, Sinopharm Chemical Reagent Co., Ltd, 0.2 mm in diameter) with fixed length (10 mm exposed) were used directly after being washed with deionized (DI) water and ethyl alcohol several times and dried. The Au modification sample, T-Ag, is prepared by immersing the silver wire into a 10 mM HAuCl_4_ solution for 10 s and then washing with DI water and ethyl alcohol.

### Characterization

The morphology was examined by field emission scanning electron microscopy (FESEM, Hitachi LEO 1530) equipped with an EDS. The XRD pattern was collected through a Bruker D8 Advance X-ray diffractometer with Cu Kα radiation (*l* = 0.15418 nm). Raman spectra were collected with a Horiba LabRAM HR Evolution system equipped with a semiconductor laser (with a wavelength of 532 nm, Laser Quantum Ltd). The *operando* Raman signal was recorded in the time mapping mode with a cyclic voltammetry test at a scan rate of 0.625 mV s^−1^. The cell used for the *operando* Raman measurement is a three-electrode system with silver wire as the working electrode, Pt mesh as the counter electrode, Hg/HgO (1 M KOH) as the reference electrode, and a glass window through which laser can be transmitted. The electrochemical measurements, including GCD and CV, are conducted using a CHI 660E workstation (Shanghai Chenhua Ltd, China) in a three-electrode system in a 1 M KOH aqueous electrolyte. The coulombic efficiency (CE) of an electrode is calculated based on the following equation: 
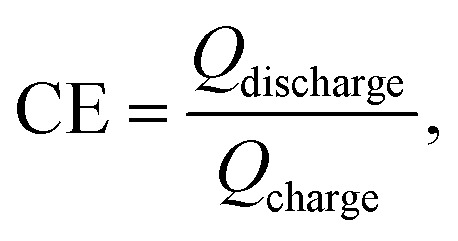
 where *Q*_discharge_ and *Q*_charge_ represent the charge flowing through the electrode during discharging and charging, respectively.

## Conclusions

Herein, the electrochemical behavior of silver in alkaline solution is well investigated. It is found that the oxidation of Ag indeed undergoes two steps through two one-electron-involved processes and Ag can be oxidized to Ag(ii)O directly at a lower potential due to the photoelectrochemical effect in the CV mode. Meanwhile in the GCD mode, except for the two well-accepted reactions of Ag to Ag_2_O and Ag_2_O to AgO, some Ag can be oxidized to AgO directly through a two-electron-transfer process in the second oxidation step, implying different activities of these reactions in the CV and GCD mode. Therefore, a possible approach to enhance the rate performance of the Ag-based electrode is proposed, *i.e.* by controlling the charge/discharge condition to avoid the formation of high resistance Ag_2_O.

During the cathodic CV sweep and galvanostatic discharge, reversible reactions take place. However, the CE is only ∼82% calculated from the CV curve at 5 mV s^−1^. Using *operando* Raman spectroscopy, it is found that the main reason for the low CE is the existence of irreversible reactions with the formation of Ag(OH)_*x*_^*n*−^. After the modification of Au, the conductivity of the electrode is improved and the formation of Ag(OH)_*x*_^*n*−^ is inhibited. The coulombic efficiency is hence improved to 95% in the CV mode and 98% in the GCD mode, indicating a promising method to enhance the reversibility of Ag-based electrochemical cells.

## Conflicts of interest

There are no conflicts to declare.

## Supplementary Material
